# Complete Genome Sequence of *Rhodococcus* sp. Strain SGAir0479, Isolated from Indoor Air Collected in Singapore

**DOI:** 10.1128/MRA.00622-19

**Published:** 2019-10-03

**Authors:** Elaine L. Oliveira, Daniela I. Drautz-Moses, Akira Uchida, Rikky W. Purbojati, Anthony Wong, Kavita K. Kushwaha, Alexander Putra, Ngu War Aung, Balakrishnan N. V. Premkrishnan, Cassie E. Heinle, Vineeth Kodengil Vettath, Ana Carolina M. Junqueira, Stephan C. Schuster

**Affiliations:** a Singapore Centre for Environmental Life Sciences Engineering, Nanyang Technological University, Singapore; b Departamento de Genética, Instituto de Biologia, Universidade Federal do Rio de Janeiro, Rio de Janeiro, Brazil; University of California, Riverside

## Abstract

The complete genome sequence of *Rhodococcus* sp. strain SGAir0479 is presented here. This organism was isolated from an air sample collected in an indoor location in Singapore. The consensus assembly generated one chromosome of 4.86 Mb (G+C content of 69.8%) and one plasmid of 104,493 bp.

## ANNOUNCEMENT

Members of the genus *Rhodococcus* are naturally present in diverse temperate and extreme environments, and they can persist and grow in highly contaminated soils and waters (1). Some strains show pathogenicity for humans, animals, and plants (2). In addition, most of the described species are capable of metabolizing a wide variety of environmental pollutants, including trichloroethene, haloalkanes, and dibenzothiophene (3–6).

*Rhodococcus* sp. strain SGAir0479 was isolated from an air sample collected in an indoor area in Singapore (1°20′42.5ʺN 103°40′44.2ʺE) using an Andersen single-stage impactor (SKC BioStage, USA) operating at 28.3 liters/min for 3 min. The air was impacted onto Trypticase soy agar (TSA) (Becton, Dickinson, USA) plates, which were then incubated overnight at 30°C. CFU were manually isolated, and strain SGAir0479 was further cultured in lysogeny broth (LB; Becton, Dickinson, USA) overnight at 30°C, followed by genomic DNA extraction with the Wizard genomic DNA purification kit (Promega, USA). Preliminary taxon identification screening was performed with Sanger sequencing using 16S rRNA universal primers 27F and 1392R (7) and subsequent BLASTn search of the sequencing result. The PacBio library was prepared with the SMRTbell template prep kit version 1.0 (Pacific Biosciences, USA) and subjected to single-molecule real-time (SMRT) sequencing on the PacBio RS II platform, which generated a total of 93,748 subreads with a combined total of 1,199,875,648 bases.

The sequencing reads were then assembled with the Hierarchical Genome Assembly Process (HGAP) version 3 (8) implemented in the PacBio SMRT Analysis 2.3.0 package. Polishing of the assembly was performed with Quiver (8). The consensus assembly generated two contigs, one chromosome of 4.86 Mb (156.5-fold coverage) and one plasmid of 104,493 bp (68.2-fold coverage). The chromosomal contig showed a mean G+C content of 69.8%.

The NCBI Prokaryotic Genome Annotation Pipeline (PGAP) version 4.2 (9) was used for genome annotation. Unless specified, all software was run using default settings. A total of 4,616 genes were predicted with 4,461 protein-coding genes (PCGs), 12 rRNA genes (4 each of 5S, 16S, and 23S rRNAs), 53 tRNAs, 3 noncoding RNAs, and 87 pseudogenes. Based on Rapid Annotations using Subsystems Technology (RAST) (10) analysis (using the ClassicRAST annotation scheme and addition of the “fix frameshifts” option), the three subsystem categories with the highest feature counts were amino acids and derivatives (488), carbohydrate metabolism (400), and cofactors, vitamins, prosthetic groups, and pigment formation (321). In this respect, at least 66 genes were found to be potentially involved in metabolism of aromatic compounds. Among these, 11 genes are part of the biphenyl degradation pathway (e.g., *bphC*, *bphD*, *bphI*, and *bphj2*) previously described to be involved in polychlorinated biphenyl (PCB) degradation (6, 11). The PCB degradation is a particular metabolic capability which may provide this microorganism with the ability to perform aerobic bioremediation (12).

Average nucleotide identity (ANI) analysis (13) showed that strain SGAir0479 has only 84.0% sequence identity to the genome of the closest species, Rhodococcus agglutinans. These values are below the ANI criteria for accurate taxonomic classification of strain SGAir0479 on the species level (minimum of 95 to 96% identity required) (14). However, it is sufficient to show the relationship between strain SGAir0479 and the *Rhodococcus* genus. To further confirm or refute this taxonomic classification, Phyla-AMPHORA (15) was run using MarkerScanner.pl with the added DNA flag and using MarkerAlignTrim.pl with the options WithReference and OutputFormat phylip; 16S identification was also performed. The results showed 92.9% identity to Rhodococcus equi and 99.9% identity to *Rhodococcus* sp. strain DSD 51W, confirming the assignment of this organism to the *Rhodococcus* genus.

### Data availability.

The complete genome sequences of *Rhodococcus* sp. strain SGAir0479 and its plasmid have been deposited in DDBJ/EMBL/GenBank under the accession numbers CP039432 and CP039433, respectively, and in the SRA under accession number SRR9043824.
